# Childhood pneumonia in low-and-middle-income countries: An update

**DOI:** 10.1016/j.prrv.2019.06.001

**Published:** 2019-11

**Authors:** Diana Marangu, Heather J. Zar

**Affiliations:** a Department of Paediatrics and Child Health, University of Nairobi, Nairobi, Kenya; b Department of Paediatrics and Child Health and SA Medical Research Council Unit on Child and Adolescent Health, University of Cape Town, Cape Town, South Africa

**Keywords:** AAP, ambient air pollution, aP, acellular pertussis, CPAP, continuous positive airway pressure, CRP, C-reactive protein, DTP3, third dose of diphtheria, tetanus and pertussis vaccine, hMPV, human metapneumovirus, IS, induced sputum, LMIC, low-and-middle-income country, MTB, *Mycobacteria tuberculosis*, NPA, nasopharyngeal aspirate, OI, opportunistic infection, PCV, pneumococcal conjugate vaccine, PCR, polymerase chain reaction, PCP, *Pneumocystis pneumonia*, PTB, pulmonary tuberculosis, PERCH, Pneumonia Etiology Research for Child Health, RSV, respiratory syncytial virus, TB, tuberculosis, WHO, World Health Organization, Lower respiratory tract infection, Epidemiology, Aetiology, Prevention, Management

## Abstract

**Objectives:**

To review epidemiology, aetiology and management of childhood pneumonia in low-and-middle-income countries.

**Design:**

Review of published English literature between 2013 and 2019.

**Results:**

Pneumonia remains a major cause of morbidity and mortality. Risk factors include young age, malnutrition, immunosuppression, tobacco smoke or air pollution exposure. Better methods for specimen collection and molecular diagnostics have improved microbiological diagnosis, indicating that pneumonia results from several organisms interacting. Induced sputum increases microbiologic yield for *Bordetella pertussis* or *Mycobacterium tuberculosis*, which has been associated with pneumonia in high TB prevalence areas. The proportion of cases due to *Streptococcus pneumoniae* and *Haemophilus influenzae b* has declined with new conjugate vaccines; *Staphylococcus aureus* and *H. influenzae* non-type b are the commonest bacterial pathogens; viruses are the most common pathogens. Effective interventions comprise antibiotics, oxygen and non-invasive ventilation. New vaccines have reduced severity and incidence of disease, but disparities exist in uptake.

**Conclusion:**

Morbidity and mortality from childhood pneumonia has decreased but a considerable preventable burden remains. Widespread implementation of available, effective interventions and development of novel strategies are needed.

## Introduction

Globally, pneumonia is one of the major causes of death children under the age of five years. In 2015, approximately 700,000 children younger than 5 years died from pneumonia worldwide, despite general improvement in living conditions, improved nutrition and better vaccines [1]. Furthermore, pneumonia continues to be the leading cause of morbidity for young children outside the neonatal period, particularly in low-and-middle-income countries (LMICs) [2]. Understanding the current epidemiology, and diagnostic and management strategies in these settings may improve preventive, diagnostic and treatment approaches.

The aim of this paper was to review the recent literature on (1) the epidemiology and aetiology of childhood pneumonia in LMICs; (2) diagnostic tools; and (3) prevention and management approaches.

## Epidemiology and aetiology

### Epidemiology

Models of childhood pneumonia from the Global Burden of Disease (GBD) in 2015 show a decreasing trend for pneumonia incidence, severe morbidity, and mortality in LMICs. Approximately 101.8 million pneumonia episodes were estimated in children under5 years in 2015; an incidence of 0.15 episodes per child year. Mortality in children <5 years decreased by 37% between 2005 and 2015, with the highest pneumonia mortality and slowest decreases reported in sub-Saharan Africa. East and South East Asia, Central Europe and tropical Latin America reported the fastest (>50%) reduction in under-5 pneumonia mortality during this period. The lowest and highest under-5 pneumonia mortalities were in Finland, estimated at 0.65 deaths per 100,000, and Somalia, estimated at 546.8 deaths per 100,000 deaths respectively [1]. Population-based surveillance data in the Gambia showed a decline in the incidence of pneumonia in children 2–11 months, and 12–23 months including radiological pneumonia by 23% and 29% respectively; pneumococcal pneumonia by 58% and 75%; and hypoxic pneumonia by 57% and 72% [3]. Marked decreases in the rates of severe pneumonia in vaccinated children and in older unvaccinated populations have also been described in South Africa and Malawi following pneumococcal conjugate vaccine (PCV 7 and PCV-13) introduction [3], [4]. Similarly in Brazil, vaccination with PCV-10 was associated with a substantial reduction in all-cause pneumonia hospitalization [5], [6], [7] in the target age-groups for vaccination and in unvaccinated individuals aged 40–49 years, reflecting the herd protection provided by vaccination [5]. Recent data from a multi-country study in four LMICs showed that measles vaccine was associated with a 15–30% decrease in pneumonia in children in India and Pakistan, and remains an important intervention in the Global Action Plan for Pneumonia and Diarrhoea (GAPPD) [8].

## Aetiology

The aetiology of pneumonia has been increasingly ascribed to multiple organisms as detected by molecular testing [9] (Table 1). The increased use of pneumococcal conjugate vaccine (PCV) and *Haemophilus influenzae type b* (Hib) vaccine has changed pneumonia aetiology, with *Staphylococcus aureus* and *H. influenzae* non-type b now the commonest bacterial pathogens and viruses most common as pathogens [9], [10], [11], [12]. However, the identification of aetiological pathogens may be difficult as distinguishing colonizing from pathogenic organisms can be difficult on respiratory specimens and multiple co-pathogens are common [13].

**Table 1:** Common aetiology of pneumonia in children in LMICs.

A. Bacteria	*Staphylococcus aureus*
	*Haemophilus influenzae*
	*Streptococcus pneumoniae*
	*Mycobacterium tuberculosis*
	*Bordetella pertussis*
	*Klebsiella pneumoniae*

B. Viruses	Respiratory syncytial virus
	rhinovirus Influenza
	Human metapneumovirus
	Adenovirus
	Parainfluenza virus
	Rhinovirus
	Measles virus
	Herpes viruses – CMV, EBV

C. Fungi	*Pneumocystis jirovecii*

### Viruses

Viruses have been identified in most pneumonia episodes; however ascribing pathogenicity may be difficult unless they are invariably associated with disease. These include respiratory syncytial virus (RSV), rhinovirus influenza, parainfluenza, human metapneumovirus (hMPV), adenovirus and parainfluenza [10], [14]. Global data in 2015 showed that RSV accounted for approximately 36,000 pneumonia deaths in children under 5 years, responsible for approximately 20% of pneumonia cases; most severe cases occurred in young children or infants in LMICs [9], [15]. Influenza was responsible for approximately 10,000 childhood deaths, with an attributable incidence of 10%. Both RSV and influenza were more often associated with non-fatal pneumonia episodes than bacteria [9]. CMV-related pneumonia in HIV-infected or immunosuppressed children is a potentially lethal disease, however data from LMICs are scarce [16], [17], [18]. Although HIV-infected children with CMV-related pneumonitis on ganciclovir in an intensive care unit in South Africa had poor outcomes [18], it was effective in immunocompetent children who were CMV polymerase chain reaction (PCR) positive in bronchoalveolar lavage in a Turkish hospital [16].

### Bacteria

**Fig. 1:**
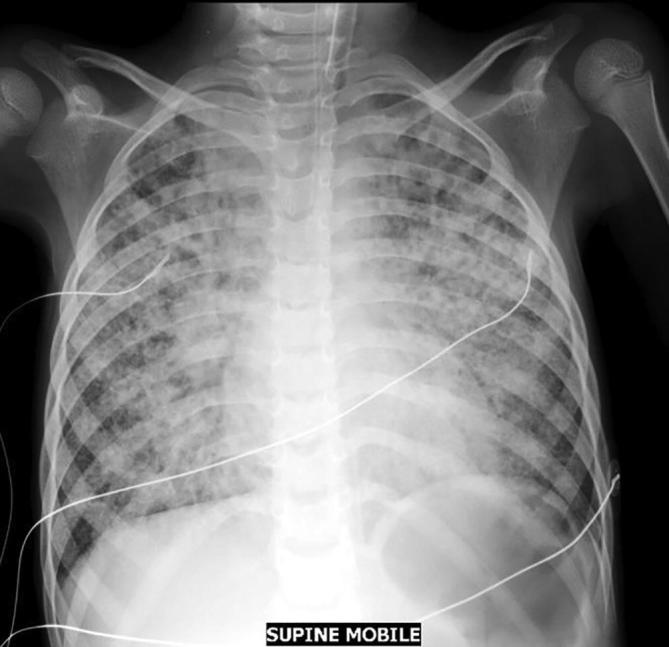
Plain chest radiograph image of a young child with pneumonia who cultured *M. tuberculosis* on an induced sputum sample.

From the 2015 GBD data, approximately 64% of pneumonia deaths in children under5 years were attributed to bacterial aetiology, specifically *Streptococcus pneumoniae* or *H. influenzae* [1]. This has changed substantially with widespread use of PCV and Hib conjugate vaccines with *S. aureus* and *H. influenzae* non-type b the commonest bacterial pathogens in the context of good coverage with these vaccines [9], [10], [12]. Other bacterial pathogens reported from LMIC settings included *Bordetella. pertussis* [10], [19], *Klebsiella pneumoniae* and *Escherichia coli* [20]. A systematic review showed that 7.5% of paediatric pneumonia was associated with culture confirmed *Mycobacterium tuberculosis* in three African studies in TB-endemic areas, which is probably a considerable underestimate of the true burden given the difficulties of obtaining microbiologic confirmation in children, Fig. 1 [21].

The burden of pertussis is high in LMICs, especially in Africa. In 2014, it was estimated there were approximately 7.8 million pertussis cases and >92,000 deaths in children <5 years in Africa [22]. Risk factors for pertussis in children include lack of vaccination, being too young to be vaccinated [10], [19], [23], [24], not receiving all three primary doses [23], [24], underweight [19], HIV exposure [19], [23] or HIV infection [23].

In high burden TB settings, pulmonary tuberculosis (PTB) is increasingly reported in children presenting with acute pneumonia, possibly in association with bacterial or viral co-infection [21], [25].

### Fungi

Pneumocystis pneumonia (PCP) was the commonest opportunistic infection in HIV-infected children before widespread use of antiretroviral therapy (ART) and effective prevention of mother-to-child transmission programmes [26], [27], [28], but has declined substantially with earlier diagnosis and treatment of HIV [26], [28]. Diagnosis may be hampered by lack of availability of PCR testing for pneumocystis – a South African study detected twice as many children with *Pneumocystis jirovecii* on PCR than on immunofluorescence or Grocott staining on respiratory samples. HIV-infected children not on ART, HIV exposure in young infants or malnutrition are risk factors for PCP that are still prevalent in sub-Saharan Africa [29].

### Risk factors

Risk factors for pneumonia incidence and severity include infancy, lack of immunization, malnutrition, chronic underlying diseases, HIV infection, HIV exposure in young infants, young maternal age, low maternal education, low socio-economic status and smoke exposure/indoor air pollution [30]. From the 2015 GBD analysis, the most important risk factors were malnutrition, household air pollution, ambient particulate matter or sub-optimal breastfeeding [1]. Complex interactions exist between risk factors for pneumonia including HIV exposure, breastfeeding, malnutrition and crowding. For example in a South African study, exclusive breastfeeding was only protective amongst HIV unexposed children, and maternal HIV infection was identified as a risk factor for pneumonia amongst exposed-uninfected infants exclusively breastfed up to 4 months, whereas crowding was a significant risk factor for well nourished children but not underweight children [31]. In LMICs, severe underweight and pallor amongst children with pneumonia were risk factors for death and may be important to consider in deciding the optimal setting for management [32]. HIV-exposed uninfected infants with pneumonia have higher rates of treatment failure and mortality in hospital during the first six months of life compared to unexposed children[33]. In 2015, 246,000 deaths were attributed to ambient air pollution (AAP) pneumonia in children under 5 years. In sub-Saharan Africa, AAP has been estimated to reduce the average life expectancy of children by 4–5 years [34] (Table 2).

**Table 2:** Risk factors for pneumonia.

**A. Host characteristics**
Infants
Lack of immunization
Lack of exclusive breastfeeding
Severe malnutrition
Chronic underlying diseases
HIV exposure in young infants
HIV infection
Low birth weight/ prematurity

**B. Socio-economic and environmental characteristics**
Young maternal age
Low maternal education
Low socio-economic status
Poor antenatal care
Tobacco smoke exposure
Indoor air pollution
Crowding

## Diagnosis

### Classification of pneumonia

In 2013, the World Health Organization (WHO) guidelines for classifying and treating childhood pneumonia were revised. Children presenting with cough or difficulty in breathing were classified into 3 diagnostic categories (pneumonia, severe pneumonia or no pneumonia) according to clinical features. Pneumonia is defined as tachypnea and/or chest indrawing in a child older than 2 months. Severe pneumonia is defined as cough or difficulty breathing with at least one of the following: (i) central cyanosis or oxygen saturation <90% on pulse oximetry, (ii) severe respiratory distress (grunting, very severe chest indrawing), or (iii) any general danger sign (inability to breastfeed or drink, lethargy or unconscious, convulsions). Children without signs of pneumonia or severe pneumonia are classified as no pneumonia: cough or cold [35]. Abnormal oxygen saturation on pulse oximetry predicts oral antibiotic failure [36]. Children with pneumonia seen at outpatient settings without the capacity to perform appropriate investigations such as pulse oximetry, nutritional assessment or HIV testing may need referral [37].

### Chest imaging

Chest X-ray remains the primary imaging modality for pneumonia (Fig. 1). The WHO developed standardized criteria for interpretation of chest radiographs in bacterial vaccine trials with three categories of radiological disease: (i) primary end-point pneumonia (end-point consolidation or pleural effusion), (ii) other infiltrate and (iii) no consolidation/infiltrate/effusion [38]. However agreement amongst readers for detection of consolidation was modest (80%, adjusted *k* = 0.60), and poor for other infiltrates (66.5%, adjusted *k* = 0.33) [39], [40]. Chest ultrasonography for paediatric pneumonia may be a rapid and safe diagnostic method [41]. Data from a recent meta-analysis show that the diagnostic accuracy of lung ultrasound had a sensitivity of 94% and specificity of 93%, against various reference tests including chest radiographs in nine studies, clinical diagnosis in four studies and computed tomography in one study. However, substantial heterogeneity exists across individual studies, and a reliable reference standard is lacking. Greater methodological rigour is needed in future studies [42].

### Blood tests

Although white blood cell count, C-reactive protein (CRP) and erythrocyte sedimentation rate are higher in children with a bacterial aetiology of pneumonia or mixed bacterial–viral aetiology in comparison to viral aetiology, these tests are non-discriminatory [43]. CRP levels of ≥40 mg/L are associated with confirmed bacterial pneumonia especially *S. pneumoniae* and *H. influenzae*, and negatively associated with RSV pneumonia. [44] Further research is needed to determine the role of CRP in discriminating confirmed bacterial pneumonia from viral pneumonia and in detecting mixed infections. Blood culture has a low yield between 3 and 7%, with a significantly higher prevalence in children with severe pneumonia, estimated at 6–14% [9], [45]. The microbial yield of blood culture in paediatric pneumonia is estimated at 2% when the blood culture volume is ≤1 ml and increases to ≥6% for volumes ≥3 ml. Conversely, antibiotic exposure reduces blood culture yield by approximately 45% [46]. *S. pneumoniae* is the most common pathogen isolated on blood culture (77%), followed by *H. influenzae* (3%) and *S. aureus* (2%) [45]. The utility of PCR for detection of pneumococcal nucleic acid (lytA gene) in blood amongst microbiologically confirmed pneumococcal pneumonia cases is limited by poor specificity (64%). Furthermore, approximately 1–10% of community controls have a positive test in blood, thus pneumococcal PCR in blood is not specific for pneumonia [47].

### Aetiological diagnosis

Nasopharyngeal aspirates (NPA) are frequently used for detection of viruses using molecular techniques and for some bacteria such as *B. pertussis*. However, distinguishing pathogenic viruses or bacteria may be difficult unless the organism is invariably associated with disease. Amongst viruses, RSV or influenza virus has been shown to be strongly associated with pneumonia in case controlled studies [10], [14]. Recent studies indicate that multiple organisms are involved in pneumonia pathogenesis and that dysbiosis in the nasopharyngeal microbiome may be key in susceptibility to pneumonia or to severe disease [48], [49]. Quantitative analysis of bacterial or viral load in the nasopharynx are not helpful for discriminating colonizing organisms from those that are disease causing although a colonization density on NPA of ≥5.9 log 10 copies/mL was predictive of Hib pneumonia [50]. Induced sputum (IS) is effective for diagnosis of pertussis, pneumocystis pneumonia or tuberculosis using molecular techniques, providing a higher yield than from NPA specimens [51], [52]. *B. pertussis* PCR on IS specimen provides confirmation earlier than NPA and increases overall diagnostic yield [23]. The yield of *P. jirovecii* in NPA, IS and non-bronchoscopic bronchoalveolar lavage samples is higher on PCR than using silver staining [53]. In the Gambia, in a study before PCV was widely available, molecular microbiological analyses of lung and pleural aspirates in children with pneumonia, identified pathogens more frequently than culture, and revealed a bacterial predominance, mainly *S. pneumoniae* or multiple microorganisms including non-typable *H. influenzae*. [12]. Although *M. tuberculosis* (MTB) PCR, specifically Xpert or Xpert Ultra is available in LMICs, IS and Xpert MTB/RIF are still infrequently available to diagnose childhood TB, with diagnosis largely based on symptom identification [54]. The sensitivity of Xpert for paediatric TB on IS is around 62% on a single specimen; with an incremental yield of up to 38% on a second specimen [55]. Ultra is an improved version of Xpert with lower limits of detection for MTB; two paediatric studies reported the sensitivity of Ultra on induced sputum of 68–77% compared to culture confirmation [56], [57].

## Prevention

Preventive interventions include childhood and maternal immunization, and optimizing nutrition, Table 3.

**Table 3:** Current preventive interventions Preventive interventions to avert pneumonia in children in LMICs.

A. Childhood immunization	Bacillus Calmette–Guérin (BCG)
	Diptheria and Pertussis (in DTP)
	Pneumococcal conjugate vaccine (PCV)
	*H. influenzae* type b vaccine Measles
	Influenza

B. Maternal immunization	Influenza
	Pertussis

C. Nutrition	Breastfeeding
	Vitamin A supplementation in measles-associated pneumonia
	†Vitamin D supplementation
	†Zinc supplementation

† Deficiency is a risk factor but currently no significant evidence of supplementation as a preventive intervention for pneumonia in well-nourished children.

### Immunization

#### Childhood immunization

While global PCV use has rapidly increased with approximately 142 countries introducing it as of 2018 [58], uptake of childhood immunization in LMICs is still sub-optimal. In 2017, PCV-13 coverage globally was estimated at 44%, with estimates for LMICs, specifically Eastern and Southern Africa at 75%, West and Central Africa at 60%, Middle East and North Africa at 40%, Latin America at 77%, South Asia at 23%, and East Asia and Pacific at 15% [59]. Considerable socio-economic inequalities in coverage of the Diptheria, Tetanus and Pertussis vaccine (DTP3), which is used as a proxy for other vaccines administered at 14 weeks including Hib and PCV, have been identified in LMIC settings [60]. In 2017, DTP3 coverage was estimated at 72% in the WHO African region [61], falling short of the WHO recommended goal of >90%. The Global Pertussis Initiative recommends that countries using whole cell pertussis continue to use it to improve primary and toddler booster vaccination coverage [62]. Significant delays exist between scheduled administration age and actual vaccination date, especially for measles vaccine where <40% are administered on schedule [63].

#### Maternal immunization

Maternal immunization against pertussis, influenza and most recently RSV has been suggested as an effective strategy to protect young infants through the first few months of life when the vulnerability to severe disease is highest [64], [65], [66]. Modelled data simulating LMIC settings showed that maternal pertussis immunization did not substantially impact on infant disease, except where mothers were not immune and eligible for multiple doses; in this group antenatal immunization reduced the incidence of infection in infants less than two months by around 30%, but there was no overall impact in infants less than one year [64]. For maternal influenza, evidence from four trials has shown that maternal immunization prevents illness in pregnant women with an overall vaccine efficacy for lab-confirmed incidence of around 30% in mothers and a vaccine efficacy of 30–63% in infants in the first 6 months of life [65]. Vaccine efficacy is lower in HIV-exposed infants [67]. The duration of protection of maternal influenza vaccination is short, being limited to the first 2–3 months of life [68]. Protection against influenza is lower when the vaccine is given late in pregnancy [69].

Maternal RSV vaccination is a novel strategy that is currently under development [66]. A model using Kenyan data showed that vaccinating pregnant women would boost maternal antibodies in infants by four more months and would potentially reduce RSV infection by >30% [70]. Given that RSV incidence and severity is highest in the first few months of life, an effective vaccine would be an important advance in this context [71]. Recent results of the first Phase 3 trial assessing the efficacy of maternal RSV vaccination, showed a 44% reduction against RSV pneumonia hospitalizations through the first six months of life. Although this trial did not meet the primary objective of significant reduction of RSV pneumonia, other pre-specified endpoints were met including a significant reduction in RSV-associated severe pneumonia, hypoxic pneumonia or hospitalisation. These results are especially important for LMICs where RSV-LRTI is more severe and may be fatal. [72].

### Nutrition

Undernutrition is a major risk factor for severe pneumonia [73] and associated mortality [74]. Prevention, and early identification and treatment of malnourished children with pneumonia is paramount including promotion of breastfeeding [75]. In children aged 2–59 months, zinc supplementation is associated with a 13% reduction in pneumonia incidence [76]. Vitamin A supplementation significantly reduces all-cause mortality and the incidence of measles in children aged 6-months to five years [77]. However, a current metanalysis shows that Vitamin A has no significant effect on the incidence of respiratory disease, hospitalization or mortality due to non-measles respiratory disease [77]. Similarly there is no evidence for the effect of vitamin D supplementation on the incidence or severity of pneumonia in children [78]. Further research is needed to determine the vitamin D concentrations associated with increased risk of pneumonia and ideal supplementation regimens [79]. However, vitamin D deficiency is significantly associated with TB infection [80] and disease in children [81], [82].

## Management

### Antibiotics

First line treatment for pneumonia is high dose oral amoxicillin (40 mg/kg/dose given twice daily) in children aged 2 months to 5 yrs for 5 days in areas with high HIV prevalence, and for 3 days in areas with low HIV prevalence. Intravenous ampicillin/benzylpenicillin and gentamicin for at least 5 days is the first line treatment recommended for children with severe pneumonia, whereas intravenous ampicillin/benzylpenicillin and gentamicin is recommended for 10 days in HIV-infected children. For HIV infected or exposed children aged from 2 months up to 1 year with severe pneumonia, cotrimoxazole is recommended as an additional treatment for suspected PCP. Prophylaxis for PCP using cotrimoxazole is recommended for all HIV-infected children and for all HIV-exposed infants from six weeks of age until HIV is confirmed negative and breast feeding has been discontinued for 6 weeks [35]; PCP prophylaxis is also highly effective for non-HIV immunocompromised children [83].

### Corticosteroids

Children hospitalized with PCP should receive oral corticosteroids within 48 h in addition to standard treatment with cotrimoxazole as this significantly reduced mortality in-hospital and 6 months after discharge [84]. Corticosteroids should also be used with tuberculosis medication to reduce nodal compression from tuberculosis if this occurs.

### Oxygen and CPAP

Oxygen saturation rates lower than 90–92% have been associated with an increased risk of death in children with pneumonia. Therefore hypoxia is an important prognostic indicator in paediatric pneumonia. However in many LMICs, the regular use of pulse oximetry and the availability of oxygen is limited [85]. An innovative low-pressure oxygen storage system piloted in Uganda, can continuously provide oxygen equivalent to the treatment of one child for 30 days despite power cuts. This system is ready for clinical field trials [86]. Similarly, a large scale implementation effectiveness trial to evaluate the feasibility and sustainability of solar powered oxygen systems in remote health centres in New Papua Guinea is ongoing [87]. In an open randomized controlled trial conducted in Bangladesh, children <5 years with severe pneumonia and hypoxaemia who received oxygen delivered via bubble continuous positive airway pressure (bCPAP) had significantly less treatment failure or death compared to those on standard low-flow oxygen [88]. The safety and efficacy of bCPAP in India has also been established [89]. Low cost bCPAP systems are being utilized in other LMIC settings. In Malawi, physicians have found bCPAP to be useful in the management of children with respiratory distress including pneumonia [90].

### Micronutrients

Vitamin A is effective for measles-associated pneumonia [91]. Given as an adjunct to the treatment of severe pneumonia in children, zinc significantly reduces mortality [92]. There is no evidence for the use of other micronutrients in the treatment of acute pneumonia in well-nourished children [93], [94]. However, nutritional support including vitamins and zinc should be given in malnourished children [35].

## Conclusion

Pneumonia remains the major cause of morbidity and mortality in children in LMICs, outside the neonatal period. With improved immunization and new conjugate vaccines, viral pathogens have been commonly reported, but multiple co-pathogens are most common. *S. penumoniae* and *H. influenzae* type b are declining with better immunisation; *S. aureus* and non-type b *H. influenzae* are becoming the commonest bacterial pathogens. TB is increasingly recognized as occurring in acute pneumonia in high burden areas. Malnutrition, HIV infection or exposure, tobacco smoke exposure and AAP are significant modifiable risk factors for pneumonia in these contexts. Available effective preventive and management strategies need to be strengthened in LMICs, while new, more accurate diagnostic and better prevention strategies are needed.

## Educational aims

The reader will be able to:

• Review the recent epidemiology and aetiology of childhood pneumonia in low-and-middle-income countries (LMICs).
• Summarize the current evidence for diagnostic tools for paediatric pneumonia in LMICs.
• Review prevention and management approaches for children with pneumonia in these settings.

## Directions for future research

Future research directions in childhood pneumonia include:

(1) comprehensive data on disease aetiology and risk factors
(2) improved implementation of effective diagnostics and interventions and
(3) innovative approaches to diagnosis, prevention and treatment including novel vaccines and rapid molecular diagnostics.

## Funding

HZ is supported by the SA Medical Research Council; HZ has received funding for studies of childhood pneumonia from the Gates Foundation Bill and Melinda Gates Foundation, USA, NIH National Institute of Health, USA, NRF National Research Foundation SA, SA-MRC SA-Medical Research Council, and NICD National Institute Communicable Diseases, SA.
